# Pathology and pathogenesis of vascular cognitive impairment—a critical update

**DOI:** 10.3389/fnagi.2013.00017

**Published:** 2013-04-10

**Authors:** Kurt A. Jellinger

**Affiliations:** Institute of Clinical Neurobiology, Medical University of ViennaVienna, Austria

**Keywords:** vascular dementia, vascular cognitive impairment, cerebral infarcts, large and small vessel disease, subcortical vascular lesions, neuropathology, pathogenic factors

## Abstract

Vascular cognitive impairment (VCI) [vascular cognitive disorder (VCD), vascular dementia] describes a continuum of cognitive disorders ranging from mild cognitive impairment (MCI) to dementia, in which vascular brain injury involving regions important for memory, cognition and behavior plays an important role. Clinical diagnostic criteria show moderate sensitivity (ca 50%) and variable specificity (range 64–98%). In Western clinical series, VaD is suggested in 8–10% of cognitively impaired elderly subjects. Its prevalence in autopsy series varies from 0.03 to 58%, with means of 8 to 15% (in Japan 22–35%). Major types of sporadic VaD are multi-infarct encephalopathy, small vessel and strategic infarct type dementias, subcortical arteriosclerotic leukoencephalopathy (SAE) (Binswanger), multilacunar state, mixed cortico-subcortical type, granular cortical atrophy (rare), postischemic encephalopathy, and a mixture of cerebrovascular lesions (CVLs). They result from systemic, cardiac and local large or small vessel disease (SVD); their pathogenesis is multifactorial. Hereditary forms of VaD caused by gene mutations are rare. Cognitive decline is commonly associated with widespread small ischemic vascular lesions involving subcortical brain areas (basal ganglia and hemispheral white matter). The lesions affect neuronal networks involved in cognition, memory, and behavior (thalamo-cortical, striato-subfrontal, cortico-subcortical, limbic systems). CVLs often coexist with Alzheimer-type lesions and other pathologies; 25–80% of elderly demented show mixed pathologies. The lesion pattern of “pure” VaD differs from that in mixed dementia (AD + CVLs) suggesting different pathogenesis of both phenotypes. Minor CVLs, except for severe amyloid angiopathy, appear not essential for cognitive impairment in full-blown AD, while both mild AD-type pathology and SVD may interact synergistically in promoting dementia. However, in a large percentage of non-demented elderly individuals, both AD-related and vascular brain pathologies have been reported. Despite recent suggestions for staging and grading CVLs in specific brain areas, due to the high variability of CVLs associated with cognitive impairment, no validated neuropathological criteria are currently available for VaD and mixed dementia. Further clinico-pathological studies and harmonization of neuropathological procedures are needed to validate the diagnostic criteria for VaD and mixed dementia in order to clarify the impact of CVLs and other coexistent pathologies on cognitive impairment as a basis for further successful therapeutic options.

## Introduction

Cerebrovascular disease (CVD) is increasingly recognized as a cause of cognitive impairment and dementia in later life either alone or in conjunction with Alzheimer disease (AD) or other pathologies. Cognitive vascular disorder describes a heterogenous group of disorders with different types of cerebrovascular lesions (CVLs) contributing to cognitive decline and finally to the development of dementia. Dementia related to vascular disorders, first described as “arteriosclerotic dementia” (McMenemey, 1961), was later replaced by terms like “multi-infarct dementia” [MID (Hachinski et al., 1974)], post-stroke dementia (Leys et al., 2005), “vascular dementia” [VaD (Garcia and Brown, 1992)], and recently by “vascular cognitive impairment” [VCI (Bowler, 2005; Moorhouse and Rockwood, 2008; Rincon and Wright, 2013)]. “Vascular cognitive disorder” [VCD (Román et al., 2004)] is a global diagnostic category encompassing all these disorders that show a presumed vascular cause, while “subcortical vascular dementia” (SVaD) means a more homogeneous syndrome (Erkinjuntti, 2002; Román et al., 2002; Bowler, 2005; Brown et al., 2006; Tomimoto, 2011).

VCI/VaD increases the morbidity, disability, and healthcare costs of the growing elderly population, and decreases their quality of life and survival (Knopman et al., 2003b; Fitzpatrick et al., 2005; Hill et al., 2005; Sicras et al., 2005). Given the substantial health and economic burden of VCI, its prevention and treatment are critical research and clinical priorities.

Consensus criteria for the clinical diagnosis of the major dementing disorders have recently been updated, e.g., the revised NIDS-ADRDA and EFNS criteria for AD (Dubois et al., 2010; Sorbi et al., 2012), the National Institute on Aging-Alzheimer's Association (NIA-AA) criteria for AD (McKhann et al., 2011; Reiman et al., 2011; Sperling et al., 2011; Sarazin et al., 2012), for Parkinson disease dementia (PDD) (Dubois et al., 2007; Emre et al., 2007), DLB (McKeith et al., 2005), frontotemporal lobe degeneration (FTLD) (Josephs et al., 2011; Seltman and Matthews, 2012), and other degenerative dementias (Karantzoulis and Galvin, 2011; Lopez et al., 2011).

Previous diagnostic criteria for VaD required the presence of memory loss and a severity of cognitive impairment sufficient to adversely affect independent functioning consistent with dementia (Chui et al., 1992; World Health Organization, 1992; Román et al., 1993; American Psychiatric Association, 1994; Kuller et al., 2005; Lopez et al., 2005). The National Institute of Neurological disorders and Stroke-Canadian Stroke Network published harmonization standards for VCI to address these potential limitations and to provide a first step toward developing diagnostic criteria for VCI (Hachinski et al., 2006). Other recent diagnostic algorithms for VCI (Zhao et al., 2010), the NINDS-AIREN DSM IV (Gorelick et al., 2011), the new EFNS-ENS guidelines for the diagnosis and treatment of disorders associated with dementia (Sorbi et al., 2012), and a proposal for new criteria for VCDs (Sachdev et al., submitted) are suggested to be suitable clinical approaches for assessing VCI patients, but await further validation.

Despite multiple attempts, no generally accepted morphologic substrates have been included in the currently used clinical diagnostic criteria for VaD (Román et al., 1993; Chui et al., 2000; Hachinski et al., 2006). In contrast to recently refined morphologic criteria for the diagnosis of AD and other degenerative dementias, no morphologic scheme for quantifying CVD associated with cognitive disturbances and no validated neuropathologic criteria for VaD/VCI have been established so far (Jellinger, 2013).

Clinico-pathologic studies reported moderate sensitivity of currently used clinical criteria (average 50–56%) and variable specificity (range 64–98%, average 87%) with variable interrater reliability (Gold et al., 2002; Chui, 2006; Bacchetta et al., 2007; Jellinger, 2007a), while the Mayo clinical criteria had 75% sensitivity and 81% specificity for autopsy-proven VaD (Knopman et al., 2003a) (Table 1). However, the demonstration of CVL by neuroimaging techniques or autopsy does not prove that they definitely cause dementia (Markesbery, 1998).

**Table 1:** **Diagnostic accuracy of possible VaD**.

**Clinical criteria**	**Pathologic diagnosis**			**Sensitivity (%)**	**Specificity (%)**
	**VaD**	**AD**	**MD**		
**(Bacchetta et al., 2007) 110 AUTOPSY CASES, MEAN AGE 94.6 ± 2.8 YEARS**					
	(*n* = 36)	(*n* = 26)	(*n* = 48)		
ADDTC possible	21	3	16	0.58	0.74
NINDS-AIREN poss.	20	3	17	0.56	0.73
HIS	20	3	22	0.56	0.66
**(Knopman et al., 2003a) 89 AUTOPSY CASES**					
Mayo clinic	12	45	11	0.75	0.81
**PREVIOUS AUTHORS (SEE Rocca and Knopman, 2003)**					
				Average	Average
				0.49 (20–89)	0.88 (64–98)

The present review, after discussing estimated prevalence and epidemiology of VaD/VCD, focuses on its major morphologic lesions, pathogenic factors, pathophysiology, and, finally, the challenges of neuropathology involved in examining the role of CVD in cognitive impairment.

## Prevalence and epidemiology

Cognitive impairment occurs after stroke in 6–41% of patients but can also arise from covert CVD (Ferrer, 2010; Pendlebury and Rothwell, 2009; Allan et al., 2011).

Given the difficulties in diagnosing VaD, considerable methodological and geographical differences, there is considerable lack of agreement about its epidemiology and prevalence.

In clinical studies, its prevalence ranges from 4.5 to 39%, in Western memory clinic- and population-based series averages 8–15.8%, with standardized incidence rates (SIR) between 0.42 and 2.68, increasing with age (Table 2).

**Table 2:** **Prevalence of VaD (clinical data) [from Jellinger (2008a)]**.

**Increase with age (years)**	**65–69**	**90+**
All dementias	0.8%	28.5%
AD (53.7 %)	0.6%	22.2%
VaD (15.8 %)	0.3%	5.2%
**Increase with age**	**65–69**	**85+**
Men	0.5%	3.6%
Women	0.1%	5.8%

In clinical studies around Europe prevalence rates of VaD between age 65–69 to 80+ years ranged from 2.2 to 16.3%, 20–40 to 200–700/100,000 and 0.7 to 6–8.1/1000 p/years (Fratiglioni et al., 2000) or 39.0/1000 p/years at age 85–88 (Aevarsson and Skoog, 1996); prevalence doubling every 5.3 years. In the US, it increased from 0.2 to 16% (Bowler, 2005) or 3.9 to 19.1% (Kuller et al., 2005), with double incidence at age 80+ in African Americans compared to Caucasians (Fitzpatrick et al., 2005), while in Japan the prevalence of VaD decreased after age 85+ (from 5.3 to 3.9%) (Kobayashi et al., 2009). In China, the prevalence for age 65+ was 4.8% for AD and 1.1% for VaD, with age-related increase of AD from 0.5–1% to 35%, while the prevalence of VaD, after an increase up to 4% in the 9th decade, decreased afterwards [see (Román, 2008)]. A meta-analysis performed on 23 studies from Europe, USA, Taiwan, China, and Japan found an increase in incidence and an exponential increase over the age of 65 years for both AD and VaD (Jorm and Jolley, 1998).

**Figure 1:**
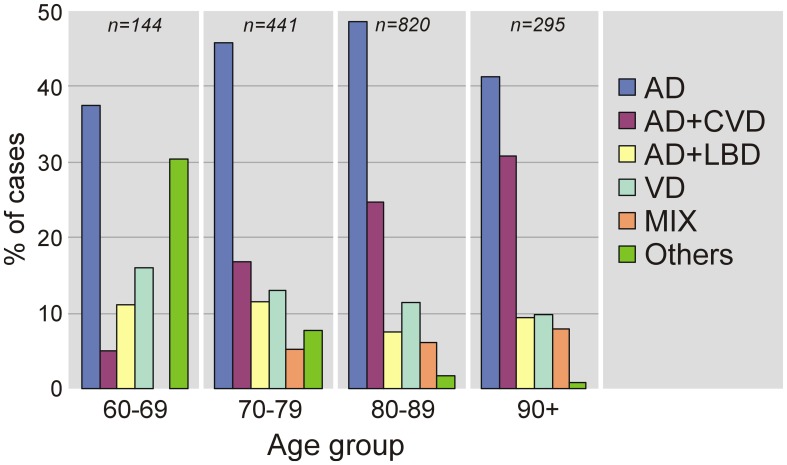
**Age-related frequency of neuropathologic types of dementing disorders.** AD, Alzheimer's disease; CVD, cerebrovascular dementia; LBD, Lewy body dementia; VaD, vascular dementia; MIX, mixed dementia (AD + cerebrovascular encephalopathy).

A review of pathologic studies shows enormous differences in the prevalence of VaD ranging from 0.03 to 85.2% with means around 11%, while in recent autopsy series from Japanese geriatric hospitals it was 23.6 to 35% (Table 3). In autopsy series of elderly subjects with and without dementia, the prevalence of “pure” VaD (without concomitant cerebral pathologies) ranged from 5 to 78%, in the oldest-old from 4.5 to 46.8% (Jellinger and Attems, 2010b). The majority of patients showed Alzheimer-related pathology, only part of them with AD alone, while mixed pathologies, i.e., AD plus cerebrovascular or Lewy pathology, were seen in 74–93% and 9–28%, respectively (Table 4). In the age group 70 to 90+, the prevalence of VaD increased from 13 to 44.8%, compared to AD (23.6–57%) and mixed dementia (2–86%) (Table 5). In a consecutive autopsy series of 1700 elderly demented, AD without concomitant pathologies or minor CVLs, and mixed dementia increased with age. Whereas the prevalence of “pure” VaD decreased after age 80+ (Figure 1).

**Table 3:** **Autopsy series showing prevalence of VaD/VCI [modified from Jellinger (2008b)]**.

1962–1990	15 studies (Europe, USA, Canada) 2784 cases
	Prevalence 2.0–85.2% (mean 24.5%)
1962–1995	(Markesbery, 1998)
	Prevalence mean 11.3%
1991–2003	(Riekse et al., 2004)
	11 studies (USA, Scandinavia, Japan) 3438 cases
	Prevalence 0.03–35% (mean 11.6%)
2004 Seattle, USA	(Snowdon and Markesbery, 1999)
	20/170 cases
	Prevalence 7%
2009 Austria	(Jellinger, 2009a): retrospective, dementia/AD
	1700/950 cases
	Prevalence 13.3/3.1% Jellinger (unpublished): prospect.
	Dementias
	300 cases
	Prevalence 9.0%
2009 Honolulu—Japan	(White, 2009)
	Demented, 183 cases
	Prevalence 33.8 %
2010 Brazil	(Nitrini et al., submitted)
	Demented, 206 cases
	Prevalence 31.8%
Japan	(Seno et al., 1999; Akatsu et al., 2002)
	122/270 cases
	Prevalence 35.0/23.6%

**Table 4:** **Frequency of mixed pathologies in autopsy series of elderly subjects**.

		Pathologies (%)^*^							
**Author**	***N***	**AD lesions**	**AD alone**	**AD + CVL**	**AD + LBD**	**VaD**	**DLB**	**FTD**	**Others**
Galasko et al., 1994	170 (d)		56.5	7.1	–	2.4	22.4		11.8
Victoroff et al., 1995	196 (d)		44.9	12.8	–	4.6	6.6		31.3
Jellinger, 1996	540 (d)		65.0	4.1	–	8.5	6.1	2.8	13.5
Nolan et al., 1998	87 (d)	100	50	34	–	–			
Lim et al., 1999	?	?	36	45	22	–			
NUN study Riley et al., 2002			57	73/93	–	–			
Akatsu et al., 2002	158 (d)		46.2	5.7		21.5	17.7	3.2	5.7
	382 (d)		41.6	11.3		3.1	22.0	4.7	17.3
	202 (d)		63.9	9.5		5.9	11.9	4.6	11.9
HAAS study Petrovitch et al., 2005	333 (235 d, 98 contr.)	<60	20	45	–	24			
MRC-CFAS (UK) Fernando and Ince, 2004	209 (48% d)	70	21	–	–	78			
Andin et al., 2005	175 (clin. VaD)	–	72	–	28				
Jellinger, 2009b (retrospective)	1700 (d)	79.5	44.8	25.3	9.0	13.3			
Jellinger, 2009b (prospective)	180 (d)	82.7	48.8	23.9	10.0	7.8			
Nitrini et al., submitted	110 (d)	43.6	28.1	15.5	(9.9)	31.8			
Brunnstrom et al., 2009	524 (d)		42.0	21.6		23.7	0.2	4.0	8.6
Schneider et al., 2007a	141 (35% d)	82.7	30	38	12	12			

AD, Alzheimer disease; CVL, cerebrovascular lesion; LBD, Lewy body disease; VaD, “pure” vascular dementia; d, demented.

* AD + other pathologies and other dementing disorders are not included.

**Table 5:** **Types of dementia in oldest-old subjects—*autopsies***.

			**% of total autopsy cases of each cohort**				
**Author**	***n***	**Mean age (years)**	**AD**	**VD**	**MIX**	**DLB**	**Others**
Mizutani and Shimada, 1992	27	(100–106)	33.3	18.5	22.0	15.0	12.0
Seno et al., 1999	122	83.6 ± 8.2	34	35	11	−	20
Akatsu et al., 2002	158	72–91	46	22	2	18	12
Riekse et al., 2004	270	80.7 ± 5.6	12 (70%F)	7 (30%F)	?	?	?
White et al., 2005	363	85.5	19	27.5	39.5^a^		14^b^
Jicha et al., 2006	34	89.0	64.7	11.7	6.0	17.6	0
Bacchetta et al., 2007	110	90+	23.6	32.7	46.4	−	−
Schneider et al., 2007a	141	86	26	16	86^c^	17	?
Grinberg et al., 2009	206	75.6	33.6	46.8	22.4	−	−
Jellinger, 2008b	1680	83.3 ± 5.6	46.4/21.0^c^	13.1	4.8	8.5	6.3
Jellinger, 2009b	300	90+	41/32	9	12	4	2
Schneider et al., 2009a							
Community cohort	194	89.8 ± 5.5	49.45	39.2	44.3	24.7	1.5
Clinical cohort	280	79.2 ± 10	66.1	17.9	27.5	21.4	6.1
Schneider et al., 2009b	179	89.7 ± 56	57.5	4.5	30.2	1.1	6.2
Brayne et al., 2009	113	91 (81–101)	67	35	26	5	?
White, 2009	183	72–90+	18.6	33.8	14.2	10.9	22.5
Jellinger and Attems, 2010a	1700	84.3 ± 5.6	45.7/25.0^c^	12.8	5.7	10.7^d^	4.2
Echavarri et al., 2012	200	78.7	26	2.0	28	0.5	47.5
Corrada et al., 2012	63	94 (90–104)	57	13.0		13.0	17.0

a 20.5% AD + CVLs, 10% Lewy neurites, 9% hippocampal sclerosis;

b no cause of dementia identified;

c AD + CVLs;

d *AD + LBs, AD, Alzheimer's disease*.

VD, vascular dementia; MIX, mixed dementia (AD + cerebrovascular encephalopathy), CVD, cerebrovascular dementia; DLB, dementia with Lewy bodies; CVLs cerebrovascular lesions; LBs, Lewy bodies.

The prevalence studies must be interpreted cautiously due to referral biases, confounding effect of their comparison by the lack of common diagnostic criteria for VaD, small numbers of very old individuals in most studies (Xuereb et al., 2000; Polvikoski et al., 2001; Kawas and Corrada, 2006; Zaccai et al., 2006), and the fact that aged subjects with and without dementia show a high frequency of mixed pathologies and comorbidities (Jellinger, 2007a,b; Schneider et al., 2007a,b; James and Schneider, 2010).

## Vessel disorders causing cerebrovascular lesions

The vessel disorders that are most frequently associated with VaD are atherosclerosis of cerebral arteries (AS), arteriosclerosis or cerebral small vessel disease (SVD), and cerebral amyloid angiopathy (CAA) (Ince, 2005; Hachinski et al., 2006; Kalaria and Erkinjuntti, 2006; Hauw et al., 2008; Ferrer, 2010; Attems et al., 2011). These vessel disorders frequently occur in the brains of elderly individuals and become more prevalent and severe with advancing age (Jellinger and Attems, 2010a). There are less common forms of CVD, including various types of vasculitis and inherited diseases that affect vessel integrity, some of which are associated with the development of dementia in the absence of AD, e.g., CADASIL.

*Atherosclerosis (AS)* is a degenerative disorder of large and medium sized arteries that leads to intima proliferation and accumulation of cholesterol within the vessel wall. These processes result in the generation and calcification of atherosclerotic plaques, that rupture frequently and induce local thrombosis. It can cause large brain infarcts, whereas embolism of atherogenic thrombi can lead to a broad variety of infarcts (Liberato et al., 2005; Grinberg and Thal, 2010).

*Small vessel disease (SVD)* comprises small vessel arteriosclerosis, lipohyalinosis, and arteriolosclerosis. These vessel wall changes are similar to that of larger blood vessels except for calcifications not seen in small arteries (Lammie, 2005; Hachinski et al., 2006). SVD can result in lacunar infarcts, microinfarcts, hemorrhages, and microbleeds (Vinters et al., 2000; Grinberg and Thal, 2010). It affects first arteries of the basal ganglia, then expands into the peripheral white matter, leptomeningeal arteries, and into thalamic and cerebellar white matter vessels, and, finally, involves brain stem arteries. Cortical vessels are usually not involved in SVD (Thal et al., 2003).

*Cerebral amyloid angiopathy (CAA)*. Sporadic CAA is characterized by the deposition of the amyloid β-protein (Aβ) in the wall of leptomeningeal and cerebral blood vessels (Attems et al., 2011). These deposits are located near the basement membrane or in the smooth muscle cell layer. CAA can lead to vessel wall rupture and hemorrhage, microbleeds, capillary occlusion, blood flow disturbances, and to microinfarcts (Thal et al., 2009; Okamoto et al., 2012). In familial forms severe CAA can be caused by Aβ as well as by the deposition of other proteins, e.g., prion protein and cystatin C, etc. (Revesz et al., 2009). CAA most frequently involves leptomeningeal and neocortical arteries, veins, and/or capillaries, later vessels in allocortical regions (hippocampus, entorhinal and cingulate cortex, amygdala). The hypothalamus and the cerebellum exhibit CAA as well, whereas blood vessels of the brainstem are involved later (Thal et al., 2003).

Intracranial hemorrhages (ICHs), including large lobar hemorrhages, deep bleeds in the basal ganglia, microscopic brain hemorrhages or cerebral micro-bleeds (CMBs), microinfarcts (seen only in microscopic sections), and subarachnoid hemorrhages may all be caused by SVD. It may also cause white matter hyperintensities (WMHs), lacunes and both ischemic and hemorrhagic infarcts. The prevalence and incidence of SVD increases with age, however, the prevalence of the lesions caused by SVD varies in the oldest old: macroscopic ICHs are comparatively rare, while microscopic hemorrhages and CMBs that are often associated with severe CAA (Tanskanen et al., 2012) are frequent. In non-demented elderly subjects, lacunes, CMBs and WMHs have been associated with cognitive decline, including reduced mental speed and impaired executive functions (Seo et al., 2007) or other neuropsychiatric symptoms. SVD is more common in subjects with AD, and might interact with the neurodegenerative changes in AD as either independent of each other (Esiri et al., 2012) or due to additive or synergistic effects on cognitive decline (Zekry et al., 2003c).

## Morphological substrates of vascular cognitive disorder

VCI/VaD is the net result of vascular lesions that lead to impairment of brain function (Jellinger, 2007a, 2008a; Ferrer, 2010; Levine and Langa, 2011). Pathologic changes in patients with VCI representing a variety of large and small cerebrovascular and ischemic lesions involving various cerebral lesions are multifold (Table 6). They include focal, multifocal, and diffuse lesions (Thal et al., 2012).

**Table 6:** **Major cerebrovascular lesions associated with cognitive impairment**.

1.	Gross large infarcts in supply territories of large cerebral arteries, in particular ACM, ACM+ACP, unilateral or bilateral
2.	Lacunes (lesions 0.5–15 mm (Ø) and multiple microinfarcts or small hemorrhages in basal ganglia, thalamus, hippocampus, basal forebrain (“strategic infarct dementia”)
3.	Multiple microinfarcts/scars in cortical border zones (“granular cortical atrophy”)—rare
4.	Pseudolaminar cortical necrosis (mainly arterial border zones)
5.	Hippocampal sclerosis
6.	White matter lesions/leukoaraiosis/Binswanger disease
7.	Combined cerebrovascular lesions

*ACM, middle cerebral artery; ACP, posterior cerebral artery*.

**Figure 2:**
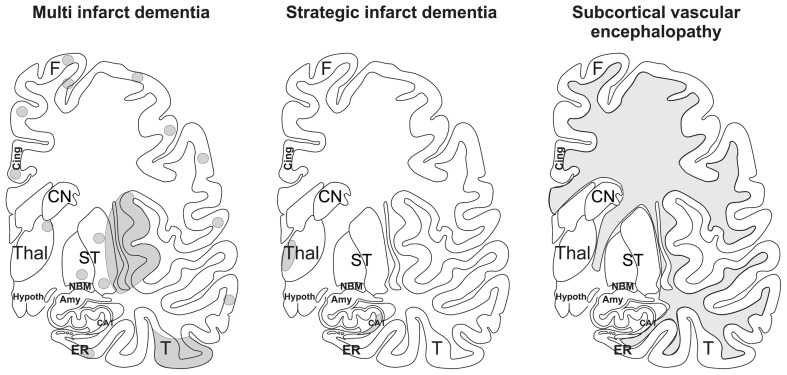
**Schematic representation of multi infarct dementia, strategic infarct dementia, and subcortical vascular encephalopathy.** The gray areas mark the regions where infarcts and white matter lesions are located. In multi infarct dementia multiple microinfarcts, lacunar infarcts, and small large infarcts are distributed all over the gray matter. Strategic infarct dementia is characterized by infarcts in strategic regions that alone explain dementia, i.e., in the hippocampal formation and in the paramedian nuclei of the thalamus. Subcortical vascular encephalopathy is characterized by confluent white matter lesions in the central and peripheral white matter. Small infarcts in subcortical brain regions may also co-occur with this type of VaD. Amy, amygdala; CA1, Ammon's horn sector CA1; Cing., cingulate gyrus; CN, caudate nucleus; ER, entorhinal cortex, F, frontal neocortex; Hypoth, hypothalamus; NBM, basal nucleus of Meynert; ST, striatum, T; temporal neocortex; Thal, thalamus. After Thal et al. (2012).

The patterns of the vascular brain lesions leading to dementia distinguish three major forms of VaD: 1. multi infarct dementia, 2. strategic infarct dementia, and 3. subcortical vascular encephalopathy (Figure 2).

1. *Multifocal lesions* include large territorial infarcts due to large vessel disease, distal field (watershed/borderzone) infarcts, mainly related to hemodynamic events and carotid artery stenosis, microinfarcts throughout the brain, often due to embolic disease, small and medium-sized lesions in subcortical functionally important brain areas, lacunes, and white matter lesions (WML). Others are subcortical arteriosclerotic leukoencephalopathy (SAE) or Binswanger's disease, resulting from chronic hypoperfusion (Ince, 2005; Farkas et al., 2006), cortical pseudolaminar necrosis due to global ischemia and hypoperfusion, hippocampal sclerosis and multiple post-ischemic lesions (Jellinger, 2007a).
2. *Focal disease* includes circumscribed unilateral or symmetric lesions, often involving functionally important brain areas and neuronal circuits (thalamus, medial temporal lobe/hippocampus, frontal and basal forebrain, etc.). They are caused by vascular and ischemic mechanisms due to large and SVD, cardiac embolic events, hemodynamic events and cerebral ischemia of various etiology, e.g., degenerative or inflammatory angiopathies, genetic arteriopathies, and other rare causes (Esiri et al., 1997; Kalaria et al., 2004; Román et al., 2004; Jellinger, 2005, 2007a; Kalimo and Kalaria, 2005; Hauw et al., 2008; Ferrer, 2010). Cardiac sources, such as atrial fibrillation and myocardial infarction, provide a source for cerebral emboli, whereas most other causes, such as hematological conditions, inflammatory angiopathies, Sneddon's disease and familial CVD, e.g., autosomal dominant arteriopathy with subcortical infarcts and leukoencephalopathy (CADASIL) (Chabriat et al., 2009) or CAA, both sporadic [see (Attems et al., 2011)] and hereditary (Kalimo and Kalaria, 2005; Revesz et al., 2009), usually cause multiple subcortical and/or cortical vascular lesions (Thal et al., 2012).

Another classification distinguishes lesions related to large and SVD (Table 7).

**Table 7:** **Classification of VaD according to major morphologic lesions (modified from Jellinger, 2007a)**.

**A. MULTIFOCAL/DIFFUSE DISEASE**
Large vessel dementia (LVD) Multiple infarct dementia (MID): Multiple large artery/borderline infarcts, cortical and subcortical, with perifocal lesions in gray and white matter. Small vessel dementia (SVD) Subcortical infarct dementia: Multiple small lacunar infarcts with perifocal lesions in white matter. “Granular atrophy” of cortex (multifocal cortical microinfarcts) Lacunes and multilacunar state Binswanger subcortical (leuko)encephalopathy Hereditary angiopathies—CADASIL (cerebral autosomal dominant arteriopathy with subcortical infarcts and leukoencephalopathy) and others Cortical plus subcortical infarct dementia: Multiple, restricted small infarcts due to: Hypertensive and arteriolosclerotic angiopathy Cerebral amyloid angiopathy, with/without hemorrhages Collagen or inflammatory vascular disease (angiitis, PCNSA, FMD) Hereditary forms of CAA Hypoperfusive, hypoxic-ischemic dementia (HHD) Incomplete white matter infarcts Anti-PL related ischemia Diffuse hypoxic-ischemic encephalopathy (cortical lacunar necrosis, post cardiac arrest, hypotension) Venous infarct dementia Large hemorrhagic, congestive symmetric infarcts due to thrombosis of the sagittal sinus or the great vein of Galen Hemorrhagic dementia Subdural hemorrhage Subarachnoid hemorrhage Intracerebral hemorrhage Multiple microbleeds, particularly subcortical
**B. FOCAL DISEASE/STRATEGIC INFARCT DEMENTIA (SID)**
Small infarcts restricted to functional important regions Mesial temporal (including hippocampal) infarcts/ischemia/sclerosis Caudate and thalamic infarcts (especially DM nucleus, bilateral lesions) Fronto-cingulate infarcts (basal forebrain, ACA) Angular gyrus infarct (dominant cerebral hemisphere - ACA and MCA territories) White matter key areas

Anti-PL, anti-phospholipid; PCNSA, primary angiitis/arteritis of the central nervous system; FMD, fibromuscular dysplasia; ACA, anterior cerebral artery; DM, dorsomedial; CAA, cerebral amyloid angiopathy.

**Figure 3:**
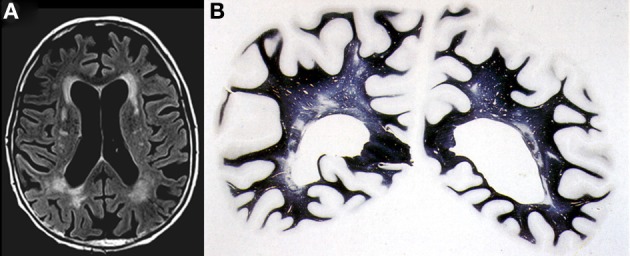
**Confluent deep white matter hyperintensities in female aged 78 years with mild cognitive impairment. (A)** T2-weighted FLAIR-MRI scan. **(B)** Multiple small areas of patchy myelin loss and lacunes in both cerebral hemispheres (Kluver-Barrera stain).

**Figure 4:**
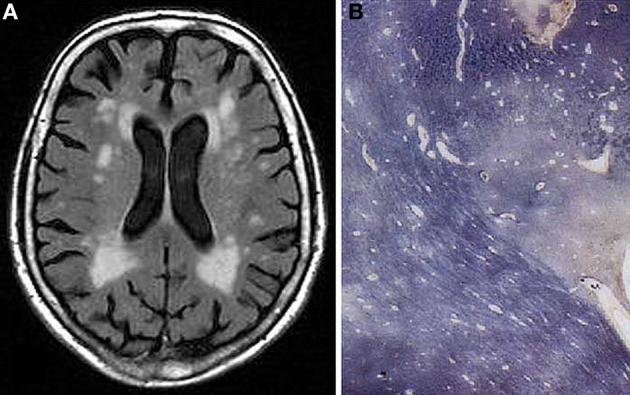
**Confluent hyperintensities in deep and periventricular white matter in 77 years old female without neuropsychiatric disorder or cognitive impairment. (A)** T2-weighted FLAIR-MRI scan. **(B)** Multiple white matter changes with patchy myelin loss, perivascular empty spaces, small lacunes, and periventrivular myelin pallor (Kluver-Barrera stain).

1. *Large vessel dementia*: Classical multi-infarct encephalopathy (MIE) is characterized by multiple large and small infarcts involving the areas of major cerebral arteries—(sub)territorial lesions of variable size, due to atherosclerosis of extra- and intracranial vessels giving rise to local thromboembolism or hypoperfusion, and cardiac sources of cerebral emboli, while inflammatory angiopathies and hereditary arteriopthies, e.g., CADASIL, more frequently cause lacunar infarcts (Ince, 2005; Hauw et al., 2008; Ferrer, 2010). Occlusion or stenosis of extracranial arteries, e.g., the internal carotid artery (ICA) and the major intracranial arteries can lead to MIE. It accounts for about 15% of VaD, the dominant hemisphere being more frequently involved. Medium-sized arteries in the leptomeninges and proximal perforating arteries can be involved. MID cannot be linked to a specific vessel disorder. Its relation to age-related vessel disorders varies and a combination of vascular lesions is frequently seen. Atherosclerosis of cerebral arteries shows a trend to more severe lesions in the circle of Willis in VaD cases than in cognitively normal controls suggesting that AS-related thrombosis and embolic events are important for this type of VaD. This trend is supported by the finding that the likelihood of dementia is increased in the presence of high-grade ICA atherosclerosis (Suemoto et al., 2011). The damage can be worse depending upon the presence of hypertension and related CVD.
2. *Small vessel disease (SVD)/microangiopathic dementia*: This type is characterized by the presence of lacunes or microinfarcts and microbleeds, predominantly involving central white matter and subcortical structures including thalamus, basal ganglia, internal capsule, brainstem, and cerebellar white matter. They are caused by hypoperfusion due to age- and hypertension-related changes of microvessels (microvascular fibrosis, stenosis and occlusion), and damage of the blood-brain barrier (BBB), etc. (Rosenberg, 2012). Lacunar infarcts are small miliary softenings from 3 to 15 mm in diameter or small cavitations that may have more than one pathologic substrate, the most significant representing small infarcts and, less frequently, healed, or reabsorbed tiny hemorrhages (Lammie, 2005; Jellinger, 2007a) that are detected by modern neuroimaging methods and upon histological examination (Figures 3, 4). Lacunes were found in 32–42% of patients studied, representing the most frequent type of CVLs (Jellinger, 2007a). Cerebral microinfarcts, minute foci with neuronal loss, gliosis, pallor, or more cystic lesions, found in all brain regions, possibly more in cerebral cortex, particularly in watershed areas, are frequent in patients with VaD (average 62%), compared with nondemented older individuals. They are an important correlate of CVL (Brundel et al., 2012; Smith et al., 2012).
  Cerebral microbleeds (CMBs) are histologically defined as blood extravasations into the perivascular and/or Virchow-Robin space usually without disruption of the surrounding tissue and/or very small intracerebral hemorrhages measuring less than 5 mm in diameter. Hemosiderin-laden macrophages and hemosiderin depositions within the perivascular space are generally considered to indicate prior CBMs (Grinberg and Thal, 2010). The walls of ruptured arterioles may show CAA related vascular damage, with thickened acellular morphology, lack of the muscular layer and Aβ deposition. In addition, ruptured microvessels affected by hypertensive angiopathy or atherosclerosis may be associated with CBMs (Vernooij et al., 2008; Poels et al., 2010; Hommet et al., 2011). CMBs potentially could also correspond to focal accumulations of hemosiderin containing macrophages in the perivascular space that are unrelated to previous bleedings. There is evidence of heme degradation activity within a surrounding inflammatory reaction with activated microglial cells, late complement activations and apoptosis (Schrag et al., 2010). The relevance of CMBs for cognitive impairment remains uncertain (Charidimou and Werring, 2012; Van der Flier and Cordonnier, 2012).
  Small vessel lesions can be distinguished according to the type and predominant location (Table 7):
  - (Multi)lacunar state with multiple cortico-subcortical microinfarcts or lacunes
  - “Strategic” infarct dementia (SID) with solitary or multiple small infarcts in functionally important brain areas (thalamus, frontocingular cortex, basal forebrain, mesial temporal area, and hippocampus) that cause cognitive deficits when damaged by CVLs) (Kalaria and Erkinjuntti, 2006; Jellinger, 2007a, 2008a; Ferrer, 2010). They can be caused by SVD and embolic events. CAA (except for familial CAA cases) is usually not associated with thalamic or hippocampal infarcts, although capillary CAA-associated vessel occlusion have been reported in the CA1-subiculum and thalamus (Thal et al., 2008, 2009).
  - Watershed or borderzone (cortico-subcortical) infarcts in cerebral convexities or end-field territories between small deep and superficial vessels (hippocampal, thalamic infarcts) caused by diminished perfusion due to atherosclerosis, prolonged episodes of hypotension.
  - Subcortical arteriosclerotic (leuko) encephalopathy type Binswanger (SAE) associated with confluent WMLs or “leukoaraoisis.” The pathology of WMLs includes a triad of demyelination, axonal loss and lacunar infarcts in the periventricular/deep and subcortical white matter ranging from dilatation of the Virchow–Robin spaces to diffuse myelin pallor and microinfarcts, usually sparing the subcortical U-fibers related to the specific pattern of vascular supply. They are caused by microvascular changes due to arteriosclerosis, hyalinosis and focal fibrinoid necrosis of vessels with and without occlusion. SVD may be associated with dilatative arteriopathy (Pico et al., 2007). These changes may not only occur in the lesions, but also in normal appearing white matter as well (Brown et al., 2007). Morphologic, experimental and molecular studies suggest that WMLs result from chronic ischemia due to hypoperfusion and disturbances of cerebral blood flow (CBF), and, alternatively recurrent edema resulting from disturbances of the BBB (Pantoni, 2002; Fernando et al., 2006). The morphologic features, pathogenesis and clinical relevance of WMLs have been reviewed (see Englund, 2004; Inzitari et al., 2007; Jellinger, 2007a; Simpson et al., 2007; Schmidt et al., 2011), but still need further validation. CADASIL can cause a similar pattern of subcortical lesions (Kalimo and Kalaria, 2005).
3. Other types of VaD include
  - *Post-ischemic encephalopathy* that can be separated into three major groups according to their predominant distribution pattern:
    - Cortical laminar necrosis and their sequelae resulting from cardiac or respiratory arrest, often occurring in arterial border zones and associated with diffuse white matter damage.
    - Multiple post-ischemic lesions after hypotension and focal narrowing of brain-feeding vessels, leading to multiple cortical and subcortical (micro)infarcts.
    - Hippocampal sclerosis (HS), a relatively common neuropathological finding (~10%) in individuals over age 85, is characterized by cell loss and gliosis that is not explained by AD. Caused by hypoxic-ischemic etiology in old subjects with cardiac failure and cerebral hypoperfusion it is often associated with dementia (Corey-Bloom et al., 1997; Attems and Jellinger, 2006; Hachinski et al., 2006; Ferrer, 2010) and has been reproduced in animal models of VaD (Hartman et al., 2005; Ishibashi et al., 2006). However, HS is also associated with a variety of neurodegenerative disorders, such as frontotemporal lobar degeneration (FTLD) and taupathies (Beach et al., 2003; Amador-Ortiz et al., 2007). A specific subtype is associated with progressed age (Nelson et al., 2011). Age at death and clinical features of HS associated with age were distinct from previously published cases of FTLD. In a recent autopsy study TDP-43 pathology was seen in 18% of cases with HS, in 35% of those with cognitive impairment and in 46% of cases with severe concomitant AS pathology (Rauramaa et al., 2011). Hence, HS may incorporate different subtypes: HS—CVD (Kril et al., 2002), HS—tau (Beach et al., 2003; Miki et al., 2009), HS—FTLD (Hatanpaa et al., 2004), and HS associated with advanced age (Nelson et al., 2011).
  - *Hemorrhagic dementia*. Primary intracerebral hemorrhages, varying in size and location, are uncommon causes of dementia, except for multiple cortical and subcortical microbleeds, usually associated with hypertension (Markesbery, 1998; Cordonnier et al., 2007; Seo et al., 2007).
  - *The role of CAA in cognitive impairment*. The majority of dementing disorders related to cerebral hemorrhages or hemorrhagic infarcts occur in sporadic and hereditary conditions associated with CAA (Zhang-Nunes et al., 2006; Revesz et al., 2009), and other hereditary forms of VaD (Kalimo and Kalaria, 2005). Sporadic CAA, for which an assessment protocol has recently been suggested (Table 8), may cause WMLs and may represent an independent risk factor for cognitive decline (Pfeifer et al., 2002; Zekry et al., 2003b; Greenberg et al., 2004; Jellinger and Attems, 2005; Chen et al., 2006). This is, however, only valid for a subgroup of demented patients lacking considerable neuritic AD pathology, whereas in the majority of cases the topographical distribution of CAA and its effect on cognitive decline are influenced by AD-pathology (Attems et al., 2007). Severe CAA is often related to cortical microinfarcts (Haglund et al., 2006), but hypertension has also an important role, and both human and experimental studies suggest that cerebrovascular effects of Aβ peptide render the brain more vulnerable to ischemic injury (Zekry et al., 2002; Iadecola, 2003). Recently CAA has been shown to be a less frequent cause of spontaneous (non-traumatic) brain hemorrhages in the aged than considered previously (Jellinger et al., 2007).

**Table 8:** **Suggested protocol for the assessment of cerebral amyloid angiopathy and concomitant vasculopathy (Chalmers et al., 2013)**.

**Score**	**Parenchymal CAA**	**Meningeal CAA**	**Capillary CAA**	**Vasculopathic score**	**Examined region**	**Vasculopathy description**
0	Absent	Absent	Absent	Absent	Frontal	FN, hemorrhage
1	Scant deposition	Scant deposition	Present	Occasional vessel	Temporal	FN
2	Some circumferential amyloid	Some circumferential amyloid		Many vessels	Parietal	FN
3	Widespread circumferential amyloid	Widespread circumferential amyloid			Occipital *median*	FN

## Pathogenic factors

Vascular risk factors (hypertension, hyperlipidemia, diabetes) and behavioral factors (obesity, physical inactivity) are associated with both CVD and, when present in mid-life, dementia (O'Donnell et al., 2010). Studies in middle-aged or older adults have found associations between VCI and hypertension (Kuller et al., 2005), hyperlipidemia (Solomon et al., 2009), diabetes (Ahtiluoto et al., 2010), obesity, and physical inactivity (Staekenborg et al., 2008). Several pathogenic mechanisms including AD, amyloid deposition, aging, atherosclerosis, and hypertension may converge to cause CVD and dementia through pathways of inflammation and oxidative stress in blood vessels (Iadecola et al., 2009). Vascular risk factors may lead to cerebrovascular dysfunction through pathways mediated by β-amyloid and the enzyme nicotinamide adenine dinucleotide phosphate (NADPH) oxidase, a major source of vascular oxidative stress. Cerebrovascular dysfunction and BBB alterations may compromise the cerebral microenvironment and increase the vulnerability of regions critical for cognition to ischemic-hypoxic brain damage leading to neuronal dysfunction and cognitive deficits (Iadecola et al., 2009). Atrial fibrillation may cause microembolic complications that lead to VCI (Puccio et al., 2009) or accelerate cognitive and functional decline (Purandare et al., 2007). Recent data may implicate clot formation and microinfarctions as mechanisms of VCI through hemostatic pathways (van Oijen et al., 2005). Other studies suggest potential roles of inflammation in VCI (Yamamoto et al., 2005).

Genetic factors may influence the development or course of VCI. ApoE ε4 and ε2 with their potential amyloidogenic role may be responsible for some of the microvascular changes in VaD (Yip et al., 2005; Baum et al., 2006). CADASIL, a genetic form of SVaD, is associated with Notch3 mutations whose location may differ by geography or demography (Chabriat et al., 2009). The identification of phenotypes that can be reliably and effectively determined in large samples of subjects is a critical challenge for genetic studies of VCI (Leblanc et al., 2006; Jones et al., 2011).

**Figure 5:**
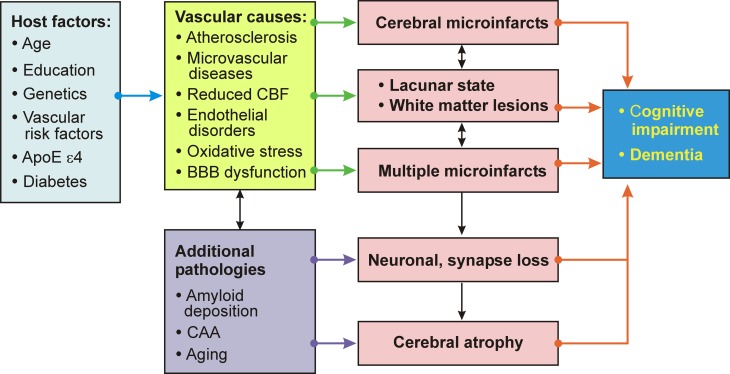
**Schematic interplay of pathogenic factors causing vascular cognitive impairment/dementia**.

Important pathogenic factors of VaD/VCD include the volume of brain destruction, its location and the number of CVLs, although the overlap between vascular and degenerative mechanisms and a frequent lack of correlation between clinical and pathology findings ware emphasized (Pantoni, 2003). The vicious circle of pathogenic factors related to VaD/VCD is summarized in Figure 5.

### Volume of brain destruction

Patients with brain tissue losses of more than 100 ml suffered from dementia and infarcts between 50 and 100 ml produced dementia less consistently, but dementia also occurred with infarcts of smaller volumes. Demented patients showed significantly more frequent infarcts >20 ml than controls, and a difference between both groups was present at 50 ml tissue loss cut-off (Tomlinson et al., 1970). According to this study, which has never been replicated, relatively small infarcts may or may not contribute to dementia, probably depending on their location, whereas destruction of large parts of the cortex are usually followed by dementia. Therefore, the concept of strategic sites of infarcts was proposed. Macroscopic studies in VaD revealed mean volumes of infarcted brain loss of 39–47 ml (range 1–229 ml) (Zekry et al., 2003a), while others suggested that dementia is not consistently related to the volume of infarction (del Ser et al., 1990) or found only a nonsignificant trend for lobar infarcts to occupy more brain volume than in patients without dementia (Vinters et al., 2000).

### Location of vascular lesions

Location of CVLs is more important than the volume of tissue destruction. Multiple brain areas have been implicated in VaD: infarction in the dominant hemisphere or bilateral lesions with predominant involvement of the dominant hemisphere as well as those in left angular gyrus, left or bilateral ACA and PCA territories increase the risk of dementia after stroke (del Ser et al., 1990). Bilateral thalamic infarction and lacunar lesions in basal ganglia, head of the caudate nucleus and inferior genu of the anterior capsule, interrupting corticothalamic and thalamocortical pathways, have been associated with “subcortical dementia” (Erkinjuntti, 2002; Van der Werf et al., 2003; Chabriat et al., 2009). Hippocampal infarcts and sclerosis either alone or in combination with other vascular lesions have been related to dementia (Attems and Jellinger, 2006; Hachinski et al., 2006). A recent study showed correspondence between rare focal subinsular vascular lesions, WMLs and lacunes (Tomimoto et al., 2007).

### Number of cerebrovascular lesions

Although a basic concept of VaD suggests that multiple small infarcts, irrespective of their volume, can lead to intellectual decline, only few studies have addressed this problem. The mean number of infarcts in VaD was 5.8–6.7 compared to 3.2 in non-demented subjects (del Ser et al., 1990), but additional factors, such as age, degree of aging changes, extent of WMLs, medial temporal lobe atrophy, etc, are involved in determining intellectual decline (Pohjasvaara et al., 2000).

### Importance of small cerebrovascular lesions

The impact of small CVLs has been examined in several autopsy series showing that lacunes, deep and periventricular WMLs may contribute to cognitive dysfunction. Previous studies demonstrated correlations between dementia and microvascular brain damage throughout the brain, while macroscopic infarcts were more frequent in the non-demented group (Esiri et al., 1999). However, many brains showed more than one type of vascular lesion (Vinters et al., 2000). Most were associated with severe atherosclerosis, while CAA was rare. These data were confirmed in a clinico-pathologic study of 130 elderly subjects with and without dementia. Among 91 demented patients, 27% had large old infarcts, 52% multiple subcortical lacunes, 8% cortical and subcortical microinfarcts, and 4% HS. Mild cognitive impairment (MCI) subjects showed large cystic infarcts in 31%, multiple subcortical lacunes in 52, the others had multiple infarcts of various volumes, while of 20 cognitively unimpaired controls, two-thirds had cystic solitary hemispheral infarcts, and 30% multiple subcortical lacunes with preserved thalamus (Jellinger, 2008b).

The impact of small CVLs in autopsy series with vascular pathology confined to microinfarcts (Kövari et al., 2004) and lacunes (Gold et al., 2005) showed a strong association between the extent of cerebral microinfarcts and cognitive findings. Lacunes, deep white matter and periventricular demyelination contributed equally to cognitive dysfunction and, contrary to earlier neuroimaging studies, the relationship between CDR scores and deep and periventricular WMLs was no longer significant after controlling for lacunes in a multivariant model (Gold et al., 2005). In both models, thalamic and basal ganglia, but not deep white matter lacunes, significantly predicted cognitive impairment (Giannakopoulos et al., 2007). Recent studies scoring the severity of SVD showed a significant correlation between SVD pathology severity and cognitive impairment (Launer et al., 2011; Smallwood et al., 2012).

A preponderance of cortical and subcortical microinfarcts was observed in a cohort of longitudinally followed autopsy cases of AD, 36 with concomitant small cerebral infarcts with volumes less than 10 ml, which had no impact on cognitive decline (Lee et al., 2000). These data, which were at variance to others suggesting a contribution to cognitive decline of CBLs with volumes of even less than 1 ml (Corbett et al., 1994), were confirmed in a larger sample showing that in AD with minor CVLs, the majority of lesions were lacunes in basal ganglia and/or white matter, and multiple microinfarcts (Jellinger, 2005).

Evaluation of the type and topographic pattern of CVLs in a large autopsy series of demented subjects, in cases with “pure” VaD, i.e., without essential concomitant Alzheimer type or other pathologies, showed a significantly higher frequency of small subcortical lesions (volume < 10 ml) representing 70% than of large infarcts (volume =10 ml) involving one or both hemispheres (32.5%) (Table 9). This pattern differed considerably from that in cases with mixed dementia (AD + vascular encephalopathy), where 56.6% revealed large, often lobar infarcts or multiple cortical and subcortical lesions larger than 5–10 mm in diameter involving one or both hemispheres, whereas lacunes and small subcortical microinfarcts were seen in only 36% (Table 10). These data suggest different pathogenic mechanisms between both types of disorders (Jellinger, 2007b; Jellinger and Attems, 2010a,b). According to the Religious Order Study, cerebral macroinfarcts independently contribute to the likelihood of dementia, but do not interact with AD pathology to increase the likelihood of dementia beyond their additive effect (Schneider et al., 2004).

**Table 9:** **Types and location of cerebrovascular lesions in *vascular dementia* (total 188, personal series)**.

**1) MULTIPLE INFARCTS (61 = 32.5%)**	
ACM bilateral	4
ACM left/right	9
ACM bilat. + ACPS/ACPD	2/1
ACM bilat. + ACP bilat.	2
ACMS + ACPS	4
ACMD + ACPD	4
ACP bilateral	3
ACP left/right	5/7
ACAS + ACMS	2
ACAD	1
multiple cortico-subcortical bilateral	12
multiple cortico-subcortical left hem.	2
**2) SAE (SUBCORTICAL) (108 = 57.4%)**	
Basal ganglia	21
Basal ganglia + white matter	31
Basal ganglia + thalamus (+ white matter)	33
Basal ganglia brainstem (+ thalamus)	23
**3) SID/STRATEGIC INFARCTS (19 = 10.1%)**	
Thalamus bilateral	9
Thalamus left	2
Thalamus + hippocampus	8

*ACA, anterior cerebral artery; ACM, middle cerebral artery; ACP, posterior cerebral artery; D, S, right, left; SAE, subcortical arteriosclerotic encephalopathy; SID, strategic infarct dementia*.

**Table 10:** **Types and location of cerebrovascular lesions in *mixed dementia* (*n* = 83; personal series)**.

**1) AD + MULTIPLE INFARCTS (47 = 56.6%)**	
ACM bilateral	7
ACM left	6
ACM right (+ lacunes basal ganglia)	3/1
ACM + ACA bilat	1
ACM + ACP left	2
ACM + ACP right	1
ACM + ACP left/right	3/3
ACM bilat. + ACPD	1
ACP bilateral	2
Multiple cortical and subcortical bilateral	13
Multiple left hemisphere	4
**2) AD + SAE (SUBCORTICAL) (33 = 39.8%)**	
Lacunes basal ganglia	15
Lacunes basal ganglia + white matter	8
Lacunes basal ganglia + thalamus	10
**3) AD + SID/STRATEGIC INFARCTS (3 = 3.6%)**	
Thalamus bilateral	2
Thalamus + hippocampus	1

*ACM, middle cerebral artery; ACP, posterior cerebral artery; SAE, subcortical arteriosclerotic encephalopathy; SID, strategic infarct dementia*.

In conclusion, subcortical lacunes and multiple disseminated microinfarcts appear to represent the most common morphologic features of VaD, while large cystic infarcts are less common (Kalaria et al., 2004; Ince, 2005; Jellinger, 2007a). This has been confirmed in animal models of VaD due to chronic hypertension (Kemper et al., 2001; Drobyshevsky et al., 2007). Brain atrophy accelerates cognitive decline in cerebral SVD (Jokinen et al., 2012).

### Cerebrovascular lesions and alzheimer disease

Recent emphasis on co-morbidity of AD and CVD, detected in 30–60% of AD brains and showing a large variety of lesions (Jellinger and Attems, 2005; Schneider et al., 2007a,b), the link between AD and atherosclerosis (Kalback et al., 2004; Ahtiluoto et al., 2010), cognitive impairment associated with CAA, present in up to 100% in AD brains (Greenberg et al., 2004; Attems et al., 2005), significant cerebral microangiopathy (Bailey et al., 2004), deficient clearance of amyloid across the BBB in AD (Zlokovic et al., 2005) and many other data indicate an association between CVD and AD. Vascular disorders are important features in chronic neurodegeneration in AD. Therefore, neurovascular dysfunction could have a major role in the pathogenesis of AD (Zlokovic et al., 2005; Stanimirovic and Friedman, 2012) that, even by some authors, has been considered a primary vascular disorder (de la Torre, 2002). The association between circle of Willis atherosclerosis and AD-type pathology provides further evidence that vascular disease and AD are interrelated and suggests that common etiologic or reciprocally synergetic pathophysiological mechanisms promote both pathologies (Yarchoan et al., 2012). Small vascular and AD-related pathologic determinants of dementia have been demonstrated in the oldest-old (Sinka et al., 2010). In elderly patients with subclinical or mild AD and little functional brain reserve (with frequent entorhinal tangles and moderate cortical plaques), either critically located CVLs or cortical watershed microinfarcts may worsen cognitive impairment due to a synergistic interaction of both pathologies (Esiri et al., 1999; Miklossy, 2003). In advanced or full-blown stages of AD, concomitant small vascular lesions do not significantly influence the overall state and progression of cognitive decline that is mainly related to the severity and extent of AD pathology overwhelming the effects of CVD (Lee et al., 2000; Jellinger, 2001; Jellinger and Attems, 2005; Chui et al., 2006; Jellinger, 2007a). However, other studies of AD cases with and without CVLs showed no significant differences in MMSE scores between groups, and no influence of the extent of vascular pathology, severity of AD pathology, or the prevalence of hypertension and myocardial infarcts was observed (Crystal and Dickson, 2002; Jellinger, 2007b). Microinfarcts are an important correlate of age-related VCI, but are not associated with an increased burden of AD pathology (Richardson et al., 2012). Increased WMLs are associated with decreased glucose metabolism and decline in executive function, but do not affect AD-specific pathology progression, suggesting that vascular contribution to dementia is probably additive although not necessarily independent of the amyloid pathway (Lo and Jagust, 2012). The severity of β-amyloid load in the brain is not significantly influenced by CVD except for a shift from Aβ-40 to Aβ-42 in the thalamus of elderly subjects, the reason of which is unknown (Aho et al., 2006). Other authors found accumulation of brain Aβ increasing with age in VaD subjects more than in elderly individuals without CVD (Lewis et al., 2006). SVD without abnormal amyloid imaging is more common than expected, and there is considerable clinical and MRI overlap in patients with SVD with and without abnormal Aβ imaging (Lee et al., 2011). In general, patients with CVD show significantly lower densities of plaques and tau pathology than “pure” AD for every given level of cognitive deficit (Zekry et al., 2002). However, others observed no major differences in neurodegenerative lesion load between AD and AD+CVLs, except when these are located in the temporal lobe and hippocampus, suggesting that this location may be important in the pathophysiology of both VaD and mixed dementia (del Ser et al., 2005; Sachdev et al., 2007). The thresholds for vascular and degenerative lesions for distinguishing “pure” VaD or AD from mixed cases have been critically discussed (Gold et al., 2007).

## Pathophysiology of VaD

VaD/VCD is a heterogeneous disorder, caused by vascular and/or ischemic lesions involving various, often functionally important brain areas and neuronal networks with deafferentation of frontal and limbic cortical structures and interruption of basal ganglia-cortical, cortico-cortical as well as ascending pathways by lesions in basal ganglia, thalamus, white matter, and subfrontal areas. The pattern of cognitive impairment is consistent with models of disturbed cortical and subcortical circuits in producing cognitive decline (Kramer et al., 2002), with interactions between subcortical lesions and changes in cortex and hippocampus (Fein et al., 2000) or correlations to frontal atrophy (Burton et al., 2003). WMLs impair frontal functions regardless of their location (Tullberg et al., 2004). They are associated with neocortical more than entorhinal and hippocampal atrophy (Du et al., 2002), that are more severe in AD than in VaD (Zarow et al., 2005), and increase the risk of dementia, particularly in patients with lacunar infarcts (Wen et al., 2004; Regan et al., 2006) and SVaD (van de Pol et al., 2011). Myelin loss in frontal lobe white matter was reduced in VaD compared to AD and DLB, which exhibited lower myelin density compared to aged controls (Ihara et al., 2010). Strategically situated small lesions that may be caused by a mixture of large and SVD, destruct thalamocortical, striatocortical and prefrontal-basal ganglia pathways, involving cognition, memory, and behavior (Loeb, 2001; Szirmai et al., 2002; Zekry et al., 2003b; Román, 2004). The association between cognitive impairment with lacunes in basal gray and white matter resulting from disruption of subcortico-frontal circuits (Reed et al., 2001; D'Abreu and Ott, 2005) has been confirmed by recent studies on the impact of subcortical lacunes/microinfarcts on cognitive dysfunction (Kövari et al., 2004; Gold et al., 2005; Giannakopoulos et al., 2007) and by experimental models of ischemia (Naritomi, 1991; Sarti et al., 2002).

Neurochemical studies in VaD showed abnormalities in key neurotransmitter systems, in particular in the basal forebrain cholinergic system related to diffuse WMLs and other vascular lesions involving the central axonal radiation fields for this projection pathway (Swartz et al., 2003), causing widespread disconnection of cholinergic projections. Since cholinergic mechanisms play a role in the regulation of CBF (Hamel, 2004; Sato et al., 2004), dysfunction of the cholinergic system may cause decreased CBF and hyopoperfusion as critical factors in the pathogenesis of VaD (Román et al., 2004; Tomimoto et al., 2005). While one study showed loss of cholinergic functions only in VaD with concomitant AD (Sharp et al., 2009), others confirmed the association of cholinergic pathways with dementia severity in subcortical VCI (Kim et al., 2012). Preservation of neurons in the cholinergic nucleus basalis in subcortical ischemic vascular disease was reported (Jung et al., 2012). Cholinergic deficits in VaD have been observed (Martin-Ruiz et al., 2000), and evidence of cholinergic changes was seen in animal models of VaD (Román and Kalaria, 2006). AD and mixed dementia (AD+VaD) usually have greater deficits of ChAT activity in the temporal cortex than age-matched controls and patients with VaD (Perry et al., 2005), and severe cholinergic deficits in frontal and temporal cortices have been reported in CADASIL (Keverne et al., 2006). Earlier studies have shown a significant reduction of synaptophysin immunoreactivity as a measure of synapse protein density in the cortex of Binswanger's disease brains (Zhan et al., 1994). There is paucity of data related to other neurotransmitter deficits in VaD, except for reduction in vasopressin and histamine due to lesions in the supraoptic and tuberomamillary nuclei (Ishunina et al., 2004).

## Conclusions and future perspectives

VCI/VaD is a heterogenic group of disorders that provides many challenges to the clinician, neuroradiologist and neuropathologist, in particular because evidence-based studies that seek to provide definite answers often lack clear definitions and validated consensus criteria of the disease (Murray et al., 2007).

Neuropathology has to describe the nature and severity of vascular pathology using harmonized morphologic procedures and criteria (Alafuzoff et al., 2012), addressing the question, whether the CVLs present in a particular brain are of sufficient magnitude and location to likely contribute or are even the sole substrate of the profile which was demonstrated clinically. Pathologic examination is necessary to: (1) confirm or detect vascular brain injury, especially for lesions that fall under the threshold of detection by neuroimaging; (2) confirm or identify the type of underlying CVLs, e.g., arteriolosclerosis, (fibro) hyalinosis, CAA; (3) ascertain the presence, type and extent of coincidental pathology. On the other hand, many elderly patients exhibit morphologic changes commonly seen in AD and VaD, but do not meet the clinical criteria of dementia (see Jellinger and Attems, 2012; Jellinger, 2013). A proposal for the assessment of key variables to define the pathology of VaD by the Newcastle group introduced a categorization of CVLs associated with cognitive impairment according to six subtypes (Kalaria et al., 2004) (Table 11). However, the existing concepts present difficulties in generalizing clinico-pathologic correlations from patient to patient (Ince and Fernando, 2003). The appreciation of the presence and extent of morphologically verified vascular lesions in cognitively impaired patients may be influenced by the heterogeneity of criteria for brain lesions, their quantification and interpretation applied in different centers, as recently demonstrated (Pantoni et al., 2006). The challenge of synthesizing a global “vascular pathology score” depends on uniform and standardized study inclusion criteria (Selnes and Vinters, 2006), and neuropathologic confirmation of a clinical diagnosis of VaD/VCD remains largely subjective in view of the fact of multiple pathologies involving the aged brain.

**Table 11:** **Newcastle categorization of the major cerebrovascular lesions associated with cognitive impairment (modified from Kalaria et al., 2004)**.

**VaD subtypes related to**	**Newcastle subtype**
Large infarct or several infarcts (>50 ml loss of tissue); multi-infarct dementia	I
Multiple small or microinfarcts (>3 with minimum diameter 5 mm); small vessel disease^*^ (involving greater than three coronal levels; hyalinization, CAA, lacunar infarcts, perivascular changes, microhemorrhages)	II
White matter lesions/leukoaraiosis/Binswanger disease	
Strategic infarcts (e.g., thalamus, hippocampus, basal forebrain)	III
Cerebral hypoperfusion (hippocampal sclerosis, ischemic–anoxic damage, cortical laminar necrosis, borderzone infarcts involving three different coronal levels)	IV
Cerebral hemorrhages (lobar, intracerebral, subarachnoidal)	V
Cerebrovascular changes with Alzheimer pathology (above Braak stage III); mixed dementia (according to the author's experience stage IV would be appropriate)	VI
Combined cerebrovascular lesions	

The age of the vascular lesion(s) should correspond with the time when disease began. Post-stroke cases are usually included in subtypes I–III.

* Subtype I may result from large vessel occlusion (atherothromboembolism), artery-to-artery embolism or cardioembolism. Subtype II usually involves descriptions of arteriosclerosis, lipohyalinosis, hypertensive, arteriosclerotic, amyloid or collagen angiopathy. Subtypes I, II, and V may result from aneurysms, arterial dissections, arteriovenous malformations and various forms of vasculitis.

Despite various proposals for a categorization of major CVLs (Román et al., 1993; Chui et al., 2000; Kalaria et al., 2004; Jellinger, 2007a), a harmonization of the criteria and techniques for the assessment of cerebral lesions of presumable/possible vascular origin in cognitively impaired is necessary (Pantoni et al., 2006; Alafuzoff et al., 2012). Due to the high variability of morphological findings and multifactorial pathogenesis of VCI, no generally accepted morphologic scheme for quantitating vascular brain injury and no validated neuropathological criteria for VaD have been established to date [see (Jellinger, 2007a,b; Román, 2008)]. The revised NIA-AA guidelines recommend reporting all macroscopic vascular brain injuries and microvascular lesions (microinfarcts/hemorrhages) in standard screening sections, multiple such lesions being associated with increased likelihood of cognitive impairment (Hyman et al., 2012; Montine et al., 2012). Another recent staging strategy proposing semiquantitative assessment of CVLs in 4 brain areas with a score ranging from I to IV/VI (Table 12) (Deramecourt et al., 2012) awaits further validation.

**Table 12:** **Staging of the cerebrovascular lesions (Deramecourt et al., 2012)**.

**Score**	**Staging**
**FRONTAL AND TEMPORAL LOBES**	
0	Normal appearance of brain, vessels, white matter, and cortex
I	Mild modification of vessel walls, perivascular spaces, or white matter
II	Moderate to severe but isolated modification of the vessel walls (arteriolosclerosis or amyloid angiopathy), usually associated with hemosiderin deposits in the perivascular spaces
III	Moderate to severe perivascular space dilatations either in the deep or the juxtacortical white matter
IV	Moderate to severe myelin loss
V	Presence of cortical microinfarcts
VI	Presence of large infarcts
**HIPPOCAMPUS**	
0	Normal appearance
I	Mild modification of vessel walls or perivascular spaces
II	Moderate to severe perivascular space dilatations
III	Presence of microinfarcts (usually in Ammon horn or the subiculum)
IV	Presence of large infarcts
**BASAL GANGLIA**	
0	Normal appearance
I	Mild modification of vessel walls or perivascular spaces
II	Moderate to severe perivascular space dilatations
III	Presence of microinfarcts
IV	Presence of large infarcts
**TOTAL VASCULAR SCORE**	
Frontal lobe +	
Temporal lobe +	
Hippocampus +	
Basal ganglia	
(/20)	

*Mixed type dementia*, being frequent in elderly demented persons, is diagnosed when a combination of several pathologies, e.g., AD with cerebrovascular and/or Lewy pathology, is present (Jellinger, 2007a; Ferrer, 2010). Vascular brain injury is commonly encountered in seniors with and without AD pathology (Ferrer, 2010; Grinberg and Thal, 2010; Jellinger, 2007a,b); but uniform and reproducible criteria are currently not available.

Further development of homogeneous and harmonized neuropathologic definitions and procedures in classifying vascular lesions and/or methods to more accurately characterize the independent severity of vascular and other brain lesions remain an important priority for the future. Development of common standards represents a first step in a process of use, validation and refinement. Universal and interinstitutional use of the same standards will help to identify individuals with cognitive impairment, will make future clinico-pathologic studies comparable, and, by integrating knowledge, will accelerate the pace of progress. An integrative, rather than a strictly taxonomic, approach to the study and elucidation of how vascular disease mechanisms contribute to the development of dementias has been proposed (Kling et al., 2013). Accurate diagnosis of both VaD/CVD and mixed dementia and further exploration of the structural correlates of cognitive impairment in order to get better insights into the impact of CVLs and their interrelationship with Alzheimer-type and other pathologies on cognitive function are among the most important challenges of modern neurosciences.

### Conflict of interest statement

The author declares that the research was conducted in the absence of any commercial or financial relationships that could be construed as a potential conflict of interest.
